# Interleukin 36 receptor-inducible matrix metalloproteinase 13 mediates intestinal fibrosis

**DOI:** 10.3389/fimmu.2023.1163198

**Published:** 2023-05-03

**Authors:** Kristina Koop, Karin Enderle, Miriam Hillmann, Laura Ruspeckhofer, Michael Vieth, Gregor Sturm, Zlatko Trajanoski, Anja A. Kühl, Raja Atreya, Moritz Leppkes, Patrick Baum, Janine Roy, Andrea Martin, Markus F. Neurath, Clemens Neufert

**Affiliations:** ^1^ First Department of Medicine, Universitätsklinikum Erlangen, Friedrich-Alexander-Universität Erlangen-Nürnberg, Erlangen, Germany; ^2^ Institute of Pathology, Klinikum Bayreuth, Friedrich-Alexander-Universität Erlangen-Nürnberg, Bayreuth, Germany; ^3^ Biocenter, Institute of Bioinformatics, Medical University Innsbruck, Innsbruck, Austria; ^4^ The Transregio 241 IBDome Consortium, Erlangen, Germany; ^5^ iPATH.Berlin, Campus Benjamin Franklin, Charité - Universitätsmedizin Berlin, Corporate Member of Freie Universität Berlin, Humboldt-Universität zu Berlin, Berlin, Germany; ^6^ Boehringer Ingelheim Pharma GmbH & Co KG, Biberach, Germany; ^7^ Staburo GmbH, München, Germany; ^8^ Boehringer Ingelheim Pharmaceuticals Inc, Ridgefield, CT, United States; ^9^ Deutsches Zentrum Immuntherapie, Erlangen, Germany

**Keywords:** il36 receptor, matrix metalloproteinase 13 (MMP13), collagenase 3, intestinal fibrosis, IL-36, αSMA+ fibroblasts, extracellular matrix (ECM), collagen type VI

## Abstract

**Background:**

Fibrostenotic disease is a common complication in Crohn’s disease (CD) patients hallmarked by transmural extracellular matrix (ECM) accumulation in the intestinal wall. The prevention and medical therapy of fibrostenotic CD is an unmet high clinical need. Although targeting IL36R signaling is a promising therapy option, downstream mediators of IL36 during inflammation and fibrosis have been incompletely understood. Candidate molecules include matrix metalloproteinases which mediate ECM turnover and are thereby potential targets for anti-fibrotic treatment. Here, we have focused on understanding the role of MMP13 during intestinal fibrosis.

**Methods:**

We performed bulk RNA sequencing of paired colon biopsies taken from non-stenotic and stenotic areas of patients with CD. Corresponding tissue samples from healthy controls and CD patients with stenosis were used for immunofluorescent (IF) staining. MMP13 gene expression was analyzed in cDNA of intestinal biopsies from healthy controls and in subpopulations of patients with CD in the IBDome cohort. In addition, gene regulation on RNA and protein level was studied in colon tissue and primary intestinal fibroblasts from mice upon IL36R activation or blockade. Finally, *in vivo* studies were performed with MMP13 deficient mice and littermate controls in an experimental model of intestinal fibrosis. Ex vivo tissue analysis included Masson’s Trichrome and Sirius Red staining as well as evaluation of immune cells, fibroblasts and collagen VI by IF analysis.

**Results:**

Bulk RNA sequencing revealed high upregulation of MMP13 in colon biopsies from stenotic areas, as compared to non-stenotic regions of patients with CD. IF analysis confirmed higher levels of MMP13 in stenotic tissue sections of CD patients and demonstrated αSMA+ and Pdpn+ fibroblasts as a major source. Mechanistic experiments demonstrated that MMP13 expression was regulated by IL36R signaling. Finally, MMP13 deficient mice, as compared to littermate controls, developed less fibrosis in the chronic DSS model and showed reduced numbers of αSMA+ fibroblasts. These findings are consistent with a model suggesting a molecular axis involving IL36R activation in gut resident fibroblasts and MMP13 expression during the pathogenesis of intestinal fibrosis.

**Conclusion:**

Targeting IL36R-inducible MMP13 could evolve as a promising approach to interfere with the development and progression of intestinal fibrosis.

## 1 Introduction

Intestinal fibrosis is a common complication in Crohn’s disease (CD) patients. It occurs mainly in patients with ileal disease localization but it can also affect other parts of the small intestine and the colon (1). Approximately 40% of CD patients develop obstructive symptoms and up to 70-80% require intestinal surgery within 20 years of diagnosis to eliminate stricturing areas (2). Fibrotic strictures are characterized by narrowing of the intestinal lumen and highlighted by transmural deposition of extracellular matrix (ECM) molecules. Obstructive symptoms due to intestinal fibrosis can also occur in the colon of individuals with CD, although they are more prevalent in the small intestine, possibly due to the smaller gut lumen there as compared to the large intestine (3). In addition, excessive ECM accumulation can also cause loss-of-function, e.g. by stiffening of the intestinal tissue (4, 5). Although the spectrum of immunosuppressants and biological therapies for patients with CD has increased over the last decade, the rate of fibrotic disease complications remains high (1). Surgical interventions including resection and strictureplasty remain common options to treat stenotic intestinal regions, whereas effective anti-fibrotic drugs are not available (3). However, a more detailed understanding of the mechanisms driving fibrogenesis in the intestine could pave the way for the development of novel medical options targeting fibrosis in the gut.

Our group recently demonstrated that IL36R signaling induced strong activation of intestinal fibroblasts and promoted chronic intestinal inflammation and fibrosis (6, 7). Correspondingly, significantly higher levels of IL36 were observed in tissues from patients with fibrostenotic CD and correlated with high numbers of activated fibroblasts (7). Moreover, attenuated inflammation and fibrosis was detected in different models of experimental fibrosis in mice upon antibody-mediated inhibition or genetic inactivation of the IL36R, which was hallmarked by reduced activation of fibroblasts and diminished accumulation of ECM, including collagen type I and VI (7). Previous work provided some evidence for mechanisms connecting IL36R signaling in fibroblasts with fibrosis such as modulation of cell proliferation and induction of pro-inflammatory and pro-fibrotic cytokines and chemokines, respectively (6, 7). In addition, IL36R activation was also demonstrated to increase the expression of collagen type VI in colon fibroblasts. However, the global picture of downstream mechanisms mediated by IL36R signaling in the context of intestinal fibrosis especially regarding ECM remodeling have remained fragmentary (7).

It is widely accepted that fibrotic remodeling of gut tissue is fueled by pro-inflammatory signaling, and a failure to resolve inflammation over a longer period of time can support an imbalance of ECM production and degradation, resulting in an accumulation of excessive amounts of ECM such as collagen types I, III, V and VI (7, 8). The main producers of ECM are αSMA+ fibroblasts when activated by various stimuli such as cytokines (e.g. IL6 and IL36), chemokines (e.g. CCL2 and CXCL1) or growth factors (e.g. EGF and FGF) (6, 7, 9, 10). In addition to *de novo* production of ECM, intestinal fibroblasts modulate fibrogenesis by secretion of matrix metalloproteinases (MMPs), endopeptidases that degrade specific ECM substrates (11). Interestingly, MMPs not only exert matrix degrading functions, but they are also regarded as regulators of inflammation, e.g. *via* the induction of proliferation of immune cells and fibroblasts (12). As a consequence, MMPs can have both pro- and anti-fibrotic functions depending on the organ-specific and model-related context of fibrosis (12). MMP13, also termed collagenase 3, belongs to the group of collagenases which enable degradation of collagen types I, II, III, fibronectin and aggrecan (13, 14). However, MMP13 was also reported to cleave non-matrix substrates such as CCL7 and pro-MMP9 (15, 16). In addition, MMP13 was shown to trim pro-tumor necrosis factor α (TNFα) into bioactive TNFα thereby suggesting gain of pro-inflammatory effector functions (17). MMP13 has been implicated in the pathogenesis of multiple disorders such as sepsis (17), joint diseases (18, 19), tumor invasion and metastasis (20, 21), and asbestos-induced lung disease (22). Mice deficient in MMP13 (*Mmp13-/-)* were initially used to study the importance of MMP13 for cartilage development (18). Based thereon, *Mmp13-/-* mice helped to analyze the dual role of MMP13 in liver (23, 24) and lung fibrosis (25, 26). Moreover, *Mmp13-/*- animals were protected in experimental models of acute intestinal epithelial barrier dysfunction including cecal ligation and puncture and acute DSS colitis which was mechanistically linked to MMP13-induced, alternative shedding of membrane bound TNFα (17). In intestinal tissue specimens from CD and ulcerative colitis (UC) patients with chronic inflammation, increased levels of MMP13 were detected at mRNA and protein level, and areas with elevated expression of MMP13 correlated with accentuated histologic inflammation (27, 28), suggesting potential involvement of MMP13 during molecular mechanisms driving intestinal fibrogenesis.

However, the contribution of MMP13 in fibrosis of the gut has remained unclear so far. Here, we have addressed the role of MMP13 in intestinal fibrosis and uncovered that MMP13 gene expression is regulated by IL36R signaling in intestinal fibroblasts and that inactivation of MMP13 can interfere with fibrosis development of the gut.

## 2 Materials and methods

### 2.1 Human samples

**Figure 1:**
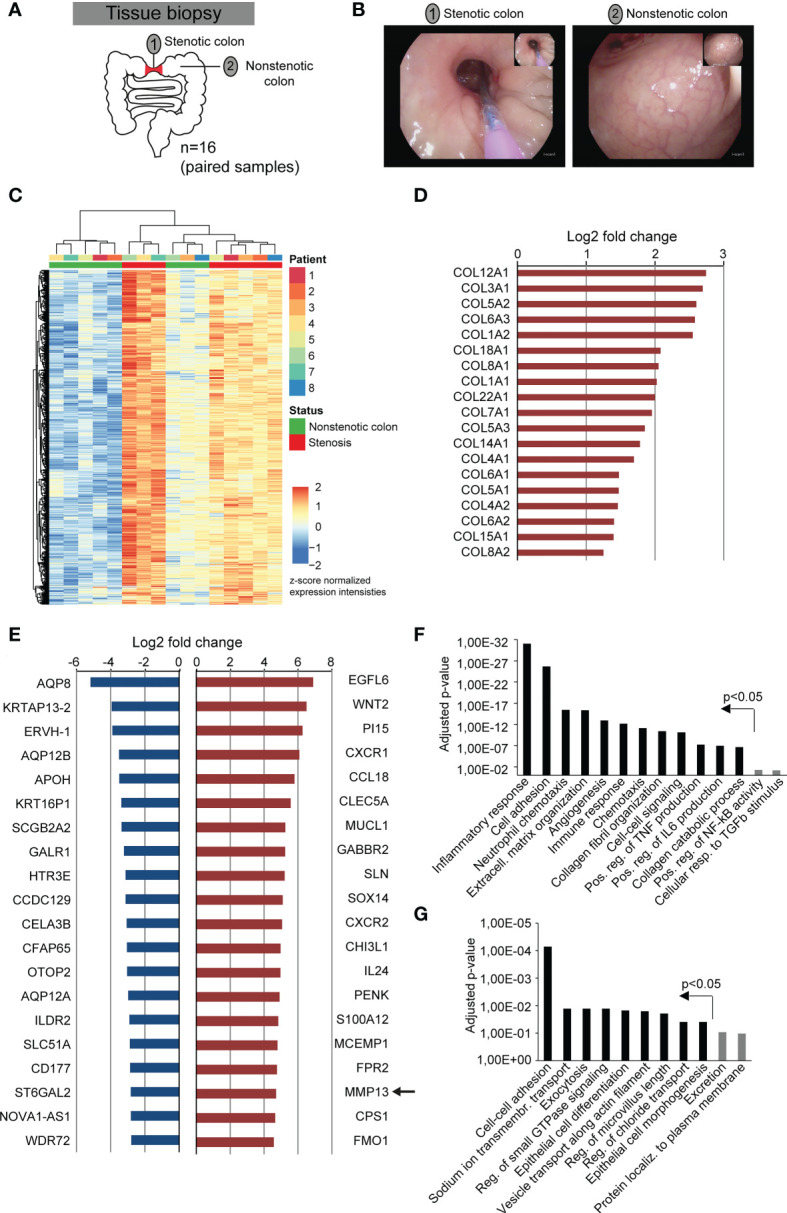
Fibrostenotic areas of CD patients are hallmarked by the expression of genes associated with extracellular matrix remodeling and inflammation. **(A)** Experimental setup shows the collection of endoscopic samples from areas of stenosis and unaffected colon during routine endoscopy of 8 different CD patients. **(B)** Representative endoscopy pictures from a stenotic area and from normal colon of the same CD patient is shown. **(C–G)** Bulk RNA-sequencing was performed of samples collected as shown in **(A)**. **(C)** Heatmap of gene expression with DEGs (p adj < 0.01, |log2fc| >1.5) is shown. **(D)** Log2 fold change of all genes encoding collagen chains that match the criteria p adj < 0.05 and |log2fc| >2 are shown. **(E)** 20 significantly differentially expressed genes (p adj < 0.05) with the highest downregulation and upregulation between non-stenotic and stenotic tissue of CD patients are shown. **(F, G)** Differentially upregulated **(F)** and downregulated **(G)** genes (p adj < 0.05, |log2fc| >1) were used for gene ontology analysis by DAVID. P-values include Benjamini-Hochberg correction.

**Figure 2:**
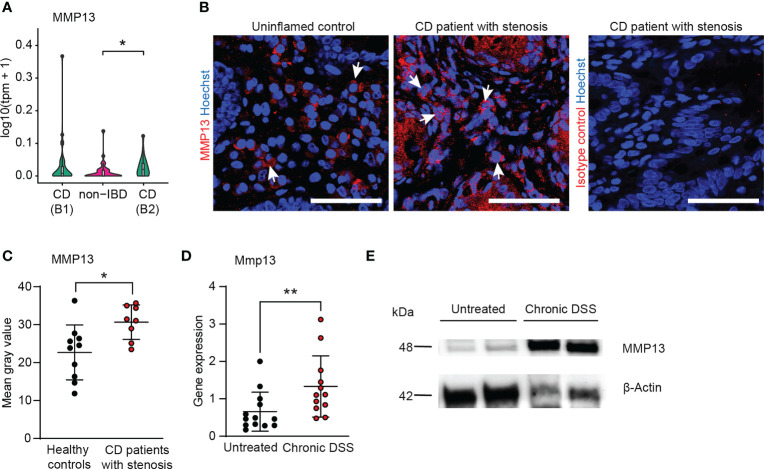
MMP13 is highly induced in human and murine fibrosis. **(A)** Bulk RNA sequencing was performed from biopsies taken during routine endoscopy from patients with CD B1 (non-stricturing, non-penetrating, Montreal classification n= 45), with CD B2 (stricturing n= 22) and non-IBD controls (n= 51) within the IBDome cohort. The expression of MMP13 is depicted. **(B, C)** Colon tissue sections from CD patients with stenosis (IF cohort) (n= 11) and from healthy controls (n= 8) were stained by immunofluorescence for MMP13 or Isotype control. The mean gray values were assessed by Image J. Representative pictures are shown. **(D)** Wildtype mice received 3 cycles of DSS and animals without treatment were used as control. Gene expression in colon tissue was analyzed at day 63 by qPCR (n= 12-13 per group). **(E)** Protein lysates (50µg) isolated from colons of untreated wildtype animals and of mice from chronic DSS-induced colitis were used for detection of MMP13 by Western Blot. β-Actin was used as loading control. Quantitative data were analyzed with Wilcoxon-Mann-Whitney test (*p>0.05, **p>0.01, two-tailed) and mean values are shown with standard deviation. Scale bar represents 50µm.

Human samples were provided by the First Department of Medicine, Friedrich-Alexander Universität Erlangen-Nürnberg (Germany), by the Charité Universitätsmedizin Berlin, Humboldt-Universität zu Berlin (Germany) and by the Institute of Pathology in Bayreuth (Germany). The analysis of intestinal tissue specimens included samples from 145 individuals (CD, n= 83; non-IBD controls, n= 62) and was based on three cohorts: samples from two cohorts were used for RNA sequencing (Stenosis cohort, **Figure 1**; **Supplemental Table 1**; IBDome cohort, **Figure 2A**; **Supplemental Table 2**) and the specimens from the third were applied to studies with immunofluorescence staining (IF cohort, **Figures 2B, C**; **Supplemental Table 3**). The analysis of human gut samples for molecular analyses was approved by the ethical review committee of Friedrich-Alexander Universität Erlangen-Nürnberg.

### 2.2 RNA sequencing

For sequencing of the stenosis cohort, paired colon biopsies were taken per patient from an unaffected region and a fibrostenotic area of 8 individuals during routine endoscopy (in sum 16 samples). The RNA was isolated and used for bulk RNA sequencing using an Illumina HiSeq 3000 (Illumina, San Diego, USA). Raw data (reads) obtained for each sample is mapped to the human reference genome hg38 (GRCh38 Ensembl v. 84) using STAR aligner v2.3. Mapped reads are QCed using RNA-SeQC v1.1.8. Gene expression intensities are represented as normalized z-scores, that were calculated based on Ensembl v84 gene annotations using RSEM version 1.2.31. Further, bamUtil version 1.0.11 and samtools version 1.1 are used for intermediate calculations like indexing of bam-files and duplicate marking. Read counts per gene are obtained using featureCounts of the subread software package version 1.5.0-p3 and further used for differential gene expression analysis. Fold changes and their respective significance are computed based on the read counts obtained for each gene using R and Bioconductor packages DESeq2 v1.18.1. Differentially expressed genes that match the criteria p < 0.05, |log2fc| >1 were used for gene ontology analysis by DAVID Bioinformatics Resources 2021 (Laboratory of Human Retrovirology and Immunoinformatics/National Institutes of Health, Bethedsa, MD) including p value correction by Benjamini-Hochberg (29). The sequencing data has been stored in the public database ArrayExpress with accession number E-MTAB-12788.

For the IBDome cohort, RNA was isolated from biopsies taken during routine endoscopy or from resected tissues at the First Department of Medicine, Friedrich-Alexander Universität Erlangen-Nürnberg (Germany) and at the Department of Gastroenterology, Infectious Diseases and Rheumatology including Clinical Nutrition at the Charité Berlin (Germany) by using a single-use biopsy forceps (Olympus). Samples were incubated in RNA protect reagent (RNAprotect Tissue Reagent, Qiagen) and stored at -80°C. For RNA isolation, one biopsy was thawed on ice and homogenized in RLT buffer (Qiagen) employing the TissueLyser LT (Qiagen). RNA was isolated, cleaned and concentrated using the RNeasy kit (Qiagen) and RNA Clean & Concentrator kit (Zymo Research). The concentration was measured at NanoDrop One/One (Thermo Fisher Scientific) and the quality (RNA integrity number, RIN) at Tape Station (Agilent). The RNA was used for bulk RNA sequencing at the NGS Competence Center Tübingen (NCCT). FASTQ files were processed with the nf-core RNA-seq pipeline version 3.4 (10.5281/zenodo.5550247) (30). In brief, reads were trimmed with TrimGalore v0.6.7 (10.5281/zenodo.5127899) and were subsequently aligned to the GRCh38 reference genome with GENCODE v33 annotation using STAR v2.7.6a (31). Read counts and transcripts per million (TPM) were quantified using Salmon v1.5.2 (32).

### 2.3 Mice

*Mmp13-/-* and *Myd88-/-* mice were previously described (18, 33). Cohoused heterozygous littermates were used as controls for *in vivo* experiments. Mice were maintained in individually ventilated cages. Animal experiments were performed in agreement with protocols approved by the government of Middle Franconia, Germany.

### 2.4 Chronic DSS-induced colitis

Chronic colon inflammation was induced by 1.5% (weight/volume) dextran sodium sulfate salt (DSS) (molecular weight 36,000-50,000 g/mol, MP Biomedicals, #160110) in the drinking water for 7 days followed by 14 days of tap water. This cycle was repeated three times (34).

### 2.5 Mini-endoscopy

Mucosal inflammation was monitored *in vivo* by the Mainz COLOVIEW^®^ System (Karl Storz, Tuttlingen, Germany) and the mucosal inflammation was graded with the murine endoscopic index of colitis severity (MEICS) as reported previously (35).

### 2.6 Purification, cultivation and stimulation of primary colon fibroblasts

Murine colon fibroblasts were enriched from colon tissue as reported previously (36). Adherent cells were cultivated with Dulbecco’s modified Eagle medium F-12 (Gibco, #31330-038) supplemented with 10% fetal calf serum (PanBiotech, #P40-37500), 1% penicillin/streptomycin (Sigma-Aldrich, #P0781), short D10. For stimulation, 0.1 Mio cells were seeded into 48 well plates in duplicates. At the next day, fresh D10 medium including IL36R ligands or PBS was added. Cells were split when their growth was confluent in culture. For *in vitro* studies, fibroblast populations from passage 5-8 were used which included < 1% of CD45+ cells as evaluated by flow cytometry.

### 2.7 Specific reagents

Recombinant human and mouse IL36R ligands (truncated proteins) were purchased from R&D Systems. IL36R stimulation by recombinant cytokines were performed with a ligand mix consisting of equal parts of IL36α (#7059-ML/CF), IL36β (#7060-ML/CF), IL36γ (#6996-IL/CF). Neutralizing rat anti-mouse IL36R chimeric antibody and isotype control antibody rat-mouse chimeric antibody were provided by Boehringer-Ingelheim (37).

### 2.8 Histopathologic analysis and evaluation of intestinal wall thickness

For histopathologic evaluation, formalin-fixed and paraffin-embedded tissue sections were stained with H&E, Masson’s Trichrome (Roth, #3459.1) and Sirius Red (Direct Red 80, Sigma-Aldrich, #365548 0.1% in picric acid, Sigma-Aldrich #P6744-1GA). Histologic changes were assessed on murine H&E sections as previously reported (38). The overall ECM content was quantified as positive pixel area by QuPath-0.3.2 (39) based on pictures of colon tissue stained with Sirius Red that were recorded with the same resolution (pixel size). Fibrotic alterations were graded from Sirius Red staining by the fibrotic score reported elsewhere (40). The fibrosis score is comprised of the level of ECM deposition within different layers of the colon wall (mucosa, submucosa, muscularis mucosa, muscularis propria) multiplied with the percent involvement of ECM distribution within the section. The thickness of the murine intestinal wall was evaluated from Sirius Red staining with NDPviewer (Hamamatsu Photonics, Hamamatsu City, Japan). The data were analyzed in a blinded fashion by two independent observers.

### 2.9 RNA isolation and qPCR

RNA isolation was performed from human biopsies, murine colon tissue and murine colon fibroblasts by the NucleoSpin RNA kit (Macherey-Nagel, #740955.250). The RNA quality and concentration was measured with a Nano Drop ND-1000 (Thermo Fisher Scientific) and 300-800ng RNA were transcribed into cDNA by SCRIPT cDNA Synthesis Kit (Jena Bioscience, #PCR-511). QPCR was performed with SSoFast EvaGreen Supermix (Biorad, #1725205) according to the manufacturer’s recommendation with the following primers: mu_Mmp13_for 5’cagtctccgaggagaaactatgat3’, mu_Mmp13_rev 5’ggactttgtcaaaaagagctcag3’. Actb (Qiagen, #249900) was used as the reference gene and samples were run on CFX96 thermal cycler (Bio-Rad Laboratories). Data analysis was performed with the Bio-Rad CFM Maestro 2.3 Software using the ΔΔCt method.

### 2.10 Immunofluorescence

Human IF stainings were performed on formalin-fixed and paraffin-embedded tissue after deparaffinization and antigen retrieval in citrate buffer (Dako, #S1699). Frozen tissue sections from murine colons were fixed in 2% PFA. Unspecific binding was blocked in all samples using 10% FCS and 1% BSA. The following antibodies were used: A647-labelled rat anti-mouse CD3 (BioLegend, #100209), eF570-labelled rat anti-mouse F480 (Invitrogen, #41-4801-82), rabbit anti-human MMP13 (Abcam, #ab39012), A488-labelled rat anti-Vimentin (Cell Signaling Technologies, #9854S), rat anti-human Pdpn (Invitrogen, #13-5381-82), A555-labelled mouse anti-αSMA (Thermo Fisher Scientific, #41-9760-82), rabbit anti-human collagen type VI (Abcam, #ab182744), rabbit polyclonal isotype control (BioLegend, #910801), rat IgG2a isotype control (BioLegend, #400512). The following secondary antibodies were used: A555-labelled anti-rabbit IgG (BioLegend, #406412), A488-labelled anti-rat IgG (BioLegend, #405418). Nuclei were counterstained with Hoechst 33342 (Life Technologies, #H3570). Stainings were recorded with Leica TCS SP5II (Wetzlar, Germany). The quantification of the mean gray value of MMP13+ staining on human tissue samples was performed with Image J (NIH, Bethesda, USA). In murine samples, IF quantification of positive cells was performed with QuPath-0.3.2 (39) and the mean gray value of collagen type VI+ staining was measured by Image J (NIH, Bethesda, USA).

### 2.11 SDS-PAGE and western blot

Proteins were isolated from murine colon tissue or cells with the M-PER extraction reagent (Thermo Fisher Scientific, #78501) supplemented with PhosSTOP (Roche, #04906845001) and cComplete ULTRA Tablets (Roche, #05892953001) according to the manufacturer’s recommendations. SDS-PAGE followed by Western Blot was performed as described previously (6). The following antibodies were used for detection: mouse anti-human/mouse MMP13 (Merck Millipore, #MAB13426), HRP-conjugated mouse anti-mouse Actin (Abcam, #ab49900), horse anti-mouse HRP (Cell Signaling, #7076S).

### 2.12 ELISA

Protein levels of MMP13 (R&D, #DY511) in cell culture supernatants were measured according to the manufacturers’ protocol.

## 3 Results

### 3.1 Expression of genes associated with inflammation and extracellular matrix remodelling is increased in stenotic areas of CD patients

To characterize molecular alterations at fibrostenotic sites in CD patients, we collected biopsies during routine endoscopy of CD patients with colonic disease manifestation. Colon biopsies were taken as paired samples from a stenotic area (n= 8) and from a non-stenotic region (n= 8) per patient (**Figures 1A, B**; **Supplementary Table 1**). Thereafter, samples (n= 16) were studied by bulk RNA sequencing. Hierarchical clustering as visualized by heatmap analysis indicated substantial similarities within the group of samples obtained from stenotic areas (**Figure 1C**). Further analysis revealed a total of 2,254 differentially expressed genes (DEG) of which 1,261 and 993 genes showed upregulation and downregulation, respectively (p adj < 0.05, |log2fc| >1). In line with expected fibrotic tissue remodelling at stenotic sites, we observed that a multitude of collagen transcripts was significantly upregulated (p adj < 0.05, |log2fc| >1) in our dataset including COL12A1, COL3A1, COL5A2, COL6A3, COL1A2 (**Figure 1D**), confirming the high quality of our fibrostenotic sample collection. Among the transcripts with the strongest upregulation within the DEGs, we noticed genes associated with ECM remodelling such as MMP13, CHI3L1, EGFL6, WNT2 (**Figure 1E**). In addition, several genes linked to proinflammatory cytokine and chemokine signalling, such as CCL18, CXCR1, CXCR2, S100A12, were also highly increased in our sample collection of stenotic vs. non-stenotic areas in the colon of CD patients, suggesting the presence of some degree of inflammation. Gene ontology (GO) analysis of DEGs further confirmed the association of fibrostenotic sites with GO terms such as extracellular matrix organization, collagen fibril organization, collagen catabolic process, inflammatory and immune response, chemotaxis, and positive regulation of TNF production (**Figure 1F**). Within the group of DEGS with the strongest downregulation, we noticed multiple membrane channels e.g. AQP8, AQP12B, OTOP2, AQP12A, SLC51A (**Figure 1E**), and GO analysis indicated enrichment of GO term such as cell-cell adhesion, sodium ion transmembrane transport, exocytosis and epithelial cell differentiation (**Figure 1G**). Initial steps to analyse gene expression in areas of fibrostenotic disease in relation to clinical parameters were done, but future analyses including a larger cohort of patients will help to understand the course of fibrostenotic disease on molecular level (**Supplemental Figures 1**, **2**).

Thus, our assumption-free approach to characterize gene expression patterns of fibrostenotic sites in the colon of CD patients using RNAseq revealed a broad variety of upregulated transcripts related to extracellular matrix remodelling and inflammation, and demonstrated strong upregulation of MMP13.

### 3.2 MMP13 is increased in CD patients with stenosis and in mice with intestinal fibrosis

On the basis of our unbiased approach with paired samples of 8 patients we had identified a group of 20 transcripts with strongest upregulation at stenotic sites (**Figure 1E**). Among these 20 genes, MMP13 seemed a particular interesting candidate molecule as it was previously connected to several mechanisms influencing gut inflammation, ECM accumulation and tissue remodeling (17, 22, 23). To further determine MMP13 expression levels in a larger cohort of patients with CD including individuals with stricturing disease phenotype (B2 Montreal classification CD) and non-stricturing, non-penetrating disease phenotype (B1 Montreal classification CD), we took advantage of the recently established IBDome database cohort (**Supplementary Table 2**) (41). Here, we noticed significant higher levels of MMP13 transcripts in the B2 disease phenotype in comparison to samples from control patients without IBD as evaluated by bulk RNA sequencing (**Figure 2A**), providing further evidence that MMP13 is associated with intestinal fibrosis. In the group of patients with B1 disease phenotype, an increase of MMP13 expression was also found, but this upregulation was not statistically significant (**Figure 2A**). Based on our findings from the sequencing data of the stenosis cohort and the IBDome collection, we performed studies for validation on protein level and compared colon tissue sections from CD patients with stenosis and healthy controls using IF. Here, we detected elevated amounts of MMP13 in colon tissue sections from CD patients with stenosis, providing additional evidence for a potential contribution of MMP13 during the pathogenesis of fibrostenotic CD (**Figures 2B, C**; **Supplementary Table 3**).

Next, we wanted to study whether MMP13 expression is also regulated in an experimental model of intestinal fibrosis. Therefore, we analyzed samples on RNA and on protein level from chronic DSS colitis, a well-established model mimicking human intestinal fibrosis (42). In accordance with our human data, we observed that the expression of Mmp13 was strongly elevated in samples with fibrosis as compared to non-fibrotic controls as evaluated by qPCR and by Western Blot (**Figures 2D, E**). In summary, our studies demonstrated an upregulation of MMP13 expression in CD patients with fibrotic stenosis as well as in mice with intestinal fibrosis.

### 3.3 IL36R stimulation induces the expression of MMP13 by intestinal fibroblasts

**Figure 3:**
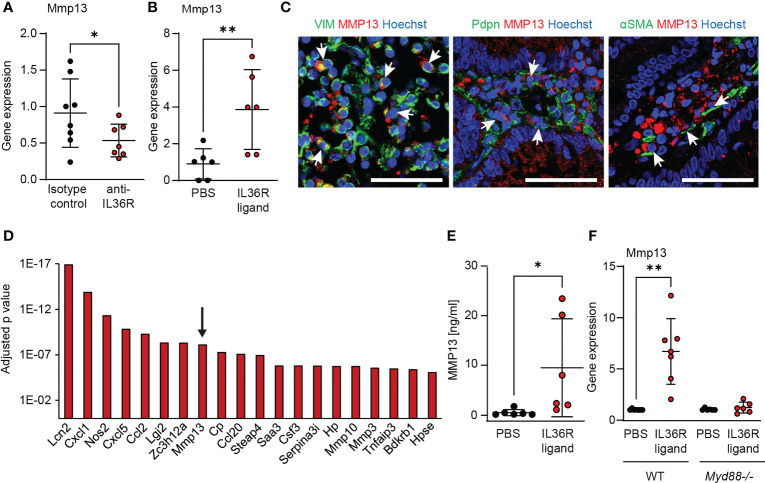
MMP13 is produced by intestinal fibroblasts upon IL36R activation. **(A)** RNA was isolated from colons of wildtype animals that were treated with an anti-IL36R antibody or an isotype control antibody (each 250µg, twice a week) during chronic DSS-induced colitis. Mmp13 expression was detected by qPCR (n= 7-8 per group). **(B)** Wildtype animals were injected with 2µg IL36R ligand mix and the expression of Mmp13 was analyzed by qPCR in colon lysates at the next day compared to the PBS injected controls. (n= 6 per group) **(C)** Representative pictures from co-stainings of MMP13 with vimentin (VIM), Pdpn or αSMA of CD patients with stenosis from the IF cohort are shown. Arrows indicate double positive cells. **(D)** Colon fibroblasts were enriched from wildtype animals and the cells were used for stimulation with IL36R ligands (100ng/ml) or PBS as control over 9 days. Fresh stimulants were given every third day. Quadruplicates were used for bulk RNA sequencing. The adjusted p value of the 20 highest regulated genes (threshold p<0.05, log2fc>2, mean count>50) is depicted. **(E)** Murine colon fibroblasts were used for chronic IL36R stimulation (100ng/ml) over 7 days. New cytokines were added every 2-3 day. Supernatants were used for detection of MMP13 protein by ELISA (n= 6). **(F)** Colon fibroblasts were enriched from untreated wildtype and *Myd88-/-* mice. The cells were stimulated for 4h with IL36R ligands (100ng/ml) or PBS (n= 6-7 per group) and gene expression was detected by qPCR. Quantitative data were analyzed by unpaired t test (*p>0.05, **p>0.01, one-tailed) and mean values are shown with standard deviation in **(A, B, E, F)** Scale bar represents 50µm.

Previous work suggested a critical role of IL36R signaling in intestinal fibrosis and stenotic IBD (7). In order to analyze if MMP13 can be a downstream target of intestinal IL36R signaling *in vivo*, we measured the expression of Mmp13 upon blockade of IL36R signaling in our model of intestinal fibrosis (34). Mice were treated intraperitoneally (i.p.) with an anti-IL36R antibody or the corresponding isotype control (each 250µg in 100µl PBS, twice a week) in a preventive setup, i.e. antibodies were administered from the beginning through the end of the experiment. Interestingly, we noticed lower levels of Mmp13 RNA transcripts in mice upon neutralization of IL36R signaling as compared to animals treated with a matching isotype control antibody (**Figure 3A**). Corresponding to the modulation of Mmp13 expression upon inhibition of IL36R signaling in the intestinal fibrosis model, we sought to analyze whether IL36R stimulation *in vivo* could also influence directly the level of Mmp13. Therefore, we performed i.p. injections with recombinant IL36R ligands vs. PBS as control into previously untreated wildtype mice and studied the expression of Mmp13 in colon tissue of these mice the next day. Strikingly, a single i.p. injection of 2 µg IL36R agonists was sufficient to cause significant upregulation of Mmp13 highlighting the role of IL36R signaling in modulating MMP13 expression *in vivo* (**Figure 3B**). Dose response studies confirmed this effect of IL36R ligands demonstrating a quantity-dependent induction of MMP13 in the colon (**Supplementary Figure 3**). Next, we wanted to identify MMP13 expressing cells in colon tissue sections from CD patients with fibrotic stenosis. To address that issue, we performed IF and detected frequent colocalization of MMP13 with vimentin+ cells, Podoplanin (Pdpn)+ cells and αSMA+ cells, suggesting stromal cells including inflammatory fibroblasts as a cellular source of MMP13 expression (**Figure 3C**).

To study the regulation of Mmp13 expression by intestinal fibroblasts upon IL36R activation, we reevaluated a previously published RNA-sequencing dataset of murine colon fibroblasts stimulated with IL36R ligands (7). The dataset addressed the modulation of global gene expression upon long-term activation of the IL36R for 9 days, while the tissue culture medium was replenished with fresh cytokines every third day. Here, Mmp13 ranked among the genes with most significant regulation and showed a log2 fold change of 2.2 (**Figure 3D**). Then, we intended to validate the data from gene transcription profiling on protein level. Hence, we performed long-term stimulation of wildtype fibroblasts with IL36 cytokines and analyzed the supernatant by ELISA. Interestingly, we observed increased levels of MMP13, thereby confirming our previous findings on a protein level (**Figure 3E**). Next, we were interested in further analysis of molecules connecting IL36R signaling with modulation of Mmp13 expression. In fact, there are multiple lines of evidence including our own studies that IL36R signal transduction involves the intracellular molecule myeloid differentiation primary response 88 (MYD88) (6). To test whether the stimulation of Mmp13 expression upon IL36R activation is also mediated *via* MYD88, we compared colon fibroblasts isolated from wildtype vs. MYD88 deficient (*Myd88*-/-) mice. Strikingly, whereas fibroblasts from wildtype mice showed a high upregulation upon IL36R stimulation, we did not detect induction of Mmp13 expression in *Myd88*-/- fibroblasts (**Figure 3F**). In sum, our mechanistic studies *in vivo* and *in vitro* evidenced the regulation of MMP13 expression by IL36R signaling in intestinal fibroblasts.

### 3.4 Defective MMP13 enzyme activity leads to decreased fibrosis in experimental fibrosis

**Figure 4:**
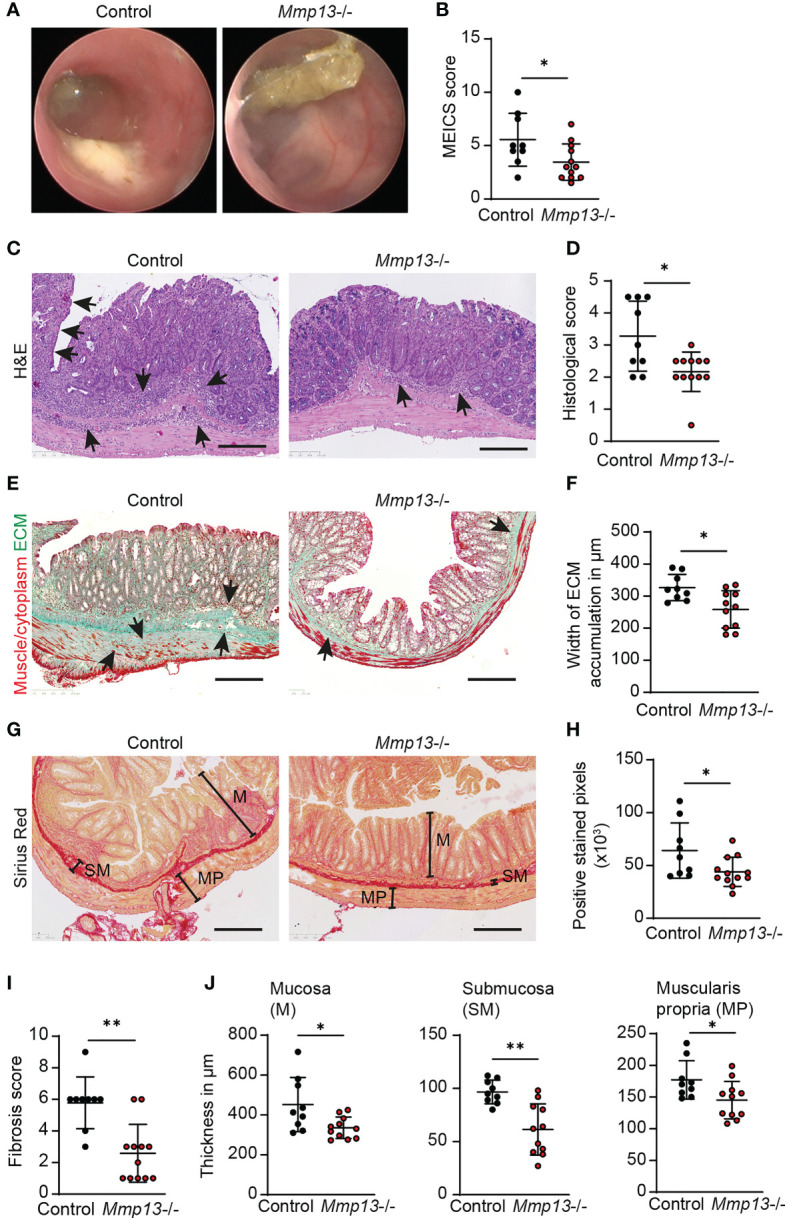
*Mmp13* deficient mice show reduced fibrosis during experimental colitis. **(A, B)** Chronic DSS-induced colitis with 3 repeated cycles of DSS administration in the drinking water was performed with *Mmp13*-/- and heterozygous littermate controls. **(A)** Colonoscopy was performed at day 63 and **(B)** the murine endoscopic index of colitis severity (MEICS) was used to score the mucosal inflammation (n= 9-11 per group). **(C, D)** Distal colon sections from *Mmp13-/-* mice vs controls from chronic DSS colitis were used for H&E stainings. Histopathological scoring of H&E stained colon sections was performed at day 63. Arrows highlight immune cell infiltration and erosion of the IEC layer. **(E, F)** Sections of distal colon tissue from *Mmp13*-/- and controls from **(A)** was used for Masson’s trichrome staining. Arrows indicate the accumulation of extracellular matrix. The width of ECM accumulation reflects the thickness of the submucosa, muscularis mucosa and muscularis propria. **(G, H)** 3 repetitive cycles of DSS were administered to *Mmp13*-/- and littermate controls. Distal colon tissue of these mice was used for Sirius Red staining. Based on the Sirius Red stainings, the amount of ECM was quantified as positive stained pixels by Qupath. **(I)** The fibrosis scoring including the distribution of ECM within the colon wall as well as the percent involvement of the tissue was assessed from colon tissue of control vs. *Mmp13*-/- mice from chronic DSS colitis, that was stained with Sirius Red. **(J)** Colon tissue of *Mmp13*-/- mice and littermate controls, that was stained for Sirius Red, was used for measurement of the thickness of the mucosa (M) (maximal width), submucosa (SM), and muscularis propria (MP) as indicated in **(G)** Quantitative data were analyzed by with Wilcoxon-Mann-Whitney test (*p>0.05, **p>0.01, two-tailed) and mean values are shown with standard deviation. Scale bar represents 250µm.

On the basis of our observations hitherto, we hypothesized that MMP13 could influence the development of intestinal fibrosis. To decipher the role of MMP13 during fibrogenesis *in vivo*, we planned experiments in mice with defective MMP13 activity (*Mmp13-/-*) (18). *Mmp13-/-* mice and littermate controls underwent chronic DSS colitis which included 3 cycles consisting of DSS in drinking water for 7 days followed by tap water for 14 days per cycle (34). Mice were evaluated for signs of inflammation by mini-endoscopy at day 63. Here, we detected reduced mucosal inflammation by mini-endoscopy in *Mmp13-/-* animals as compared to littermate controls (**Figures 4A, B**). Next, intestinal tissue was harvested, and we performed H&E staining on distal colon tissue sections. In line with our endoscopic findings, histopathological analysis revealed reduced inflammation including diminished epithelial cell erosions and immune cell infiltration in *Mmp13-/-* mice as compared to controls (**Figures 4C, D**). Then, we focused our analysis on alterations associated with intestinal fibrosis. Accordingly, we investigated into the accumulation of ECM in colon tissue sections by Masson’s trichrome staining and detected lower amounts of ECM in *Mmp13-/-* animals as compared to littermate control mice (**Figures 4E, F**). In addition, we quantified and characterized intestinal fibrosis using Sirius Red staining, which helped to visualize the distribution of ECM in various colon tissue layers including mucosa, submucosa, and muscularis propria (**Figure 4G**). Notably, we detected a significant lower overall accumulation of ECM in *Mmp13-/-* vs. control mice as reflected by the quantification of positive pixels in the Sirius Red staining and the fibrosis score (**Figures 4H, I**). To further determine the localization of the ECM, we measured the thickness of individual tissue layers of the bowel wall. In line with our previous findings, we detected a significant decrease of ECM accumulation in the mucosa, the submucosa and the muscularis propria in *Mmp13-/-* mice as compared to controls, suggesting that MMP13-related modulation of intestinal fibrosis in mice is a rather general phenomenon and not limited to a certain layer (**Figure 4J**).

**Figure 5:**
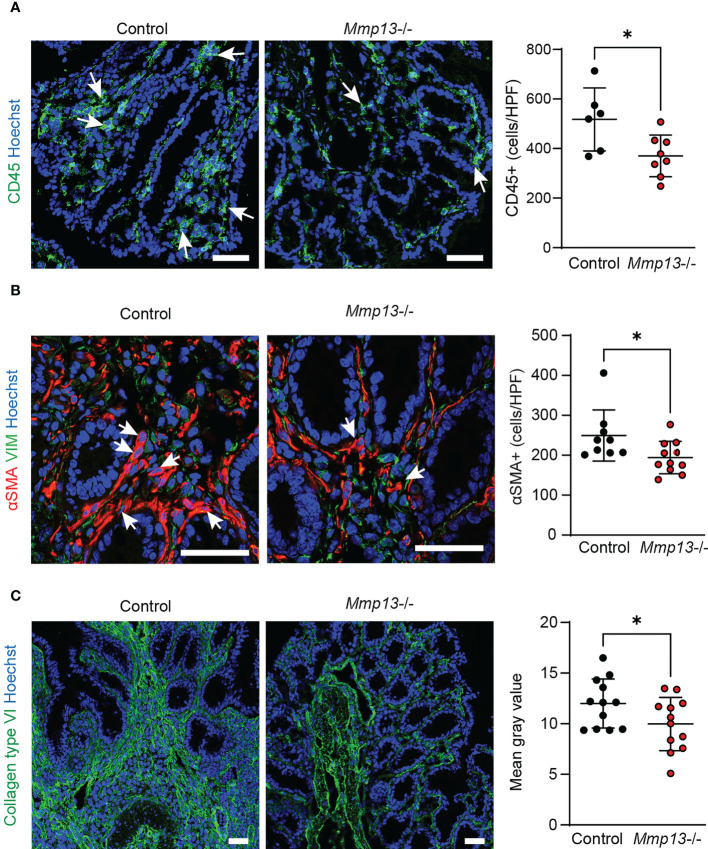
*Mmp13* deficient mice are characterized by diminished numbers of immune cells, fibroblasts and reduced deposition of collagen type VI in experimental fibrosis. *Mmp13*-/- mice or littermate controls were used for 3 repeated cycles of DSS administration in the drinking water followed by 14 days of recovery with water. At day 63, colon tissue was harvested and used for IF studies. **(A)** Immune cells were detected by staining for CD45. (n= 6-8 per group). **(B)** Activated fibroblasts were stained for αSMA. (n= 9-11 per group). **(C)** Collagen type VI was stained in colon tissue sections from *Mmp13-/-* vs controls (n= 7-11 per group). Quantitative data were analyzed by unpaired t test (*p>0.05, one-tailed) and mean values are shown with standard deviation. Positive cells were quantified by Qupath **(A, B)** and the mean gray values of collagen type VI **(C)** was assessed by Image J Arrows indicate positive cells. Scale bar represents 50µm.

On the basis of our histopathological analysis, we performed further characterization of *Mmp13-/-* mice compared to littermate controls by IF in colon tissue from chronic DSS colitis. In accordance with the analysis of H&E stainings, we observed a lower accumulation of CD45+ immune cells in the tissue of *Mmp13* defective mice (**Figure 5A**). Additional IF stainings analyzed the abundance of αSMA+ cells which had previously been demonstrated to serve as main producers of ECM components in the intestine upon activation of the IL36R (7). In fact, we detected a significant lower amount of αSMA+ cells in *Mmp13-/-* vs. control mice as studied by IF and confocal microscopy (**Figure 5B**), suggesting MMP13-mediated regulation of (myo)fibroblast accumulation in intestinal fibrosis. Thereafter, we addressed the deposition of collagen type VI which had been connected to IL36R signaling during fibrogenesis before (7). We performed IF stainings of colon sections harvested from chronic DSS colitis. Interestingly, we found that accumulation of collagen type VI was markedly decreased in *Mmp13-/-* mice as compared to littermate controls (**Figure 5C**). Thus, our work demonstrated that inactivation of MMP13 resulted in an attenuation of intestinal fibrosis *in vivo* characterized by changes in cell infiltrates such as reduced numbers of αSMA+ cells and in ECM accumulation such as collagen type VI throughout the entire bowel wall.

In summary, this study is in line with a model suggesting a molecular axis involving IL36R activation in intestinal fibroblasts and MMP13 expression during the pathogenesis of intestinal fibrosis. Targeting MMP13 expression could evolve as a promising approach to alleviate the development and progression of gut fibrosis.

## 4 Discussion

Despite therapeutic advances in the medical care of patients with IBD, the incidence of fibrostenotic complications such as strictures has remained high and frequently requires surgical interventions (2). A better understanding of the molecular mechanisms promoting intestinal fibrogenesis could help to tackle this unmet clinical need and pave the way for the development of novel therapeutic options targeting fibrosis of the gut.

This study analyzed the role of MMP13 in intestinal fibrosis. Our work included the analysis of MMP13 expression in intestinal specimens from patients with CD and control individuals without IBD. In patients with CD and a fibrostenotic disease phenotype, comparative analysis of paired samples from stenotic vs. non-stenotic areas from the same individuals was performed and revealed a high differential gene expression of MMP13 at stenotic sites. This finding was paralleled by upregulation of various genes associated with ECM remodeling including genes encoding for multiple collagen subtypes related to fibrotic disease (7, 8, 43). The expression patterns of the stenotic areas did not only connect to fibrosis, but also to inflammation associated changes. Our observation underlines at a molecular level that stenotic regions in patients with CD are rarely fibrotic only, but typically include an inflammatory constituent at variable degree which is a frequent finding during endoscopy and histopathological evaluation (44).

Our analysis of the IBDome patient cohort revealed additional evidence that MMP13 is predominantly enriched in samples from patients with CD and stenotic disease phenotype (B2 Montreal classification). This finding adds to previous reports that connected MMP13 expression with intestinal inflammation in other populations of patients with CD and UC (27, 28). Correspondingly, we also noticed a trend to elevated MMP13 transcripts in CD patients with non-stricturing, non-penetrating disease phenotype (B1 Montreal classification). By contrast, we measured almost no expression of MMP13 in healthy controls at mRNA level in human tissue specimens. That was in line with studies by others that did not observe MMP13 expression in control tissue during intact intestinal homeostasis (27, 45), suggesting that MMP13 expression is driven by inflammatory and pro-fibrotic triggers in the gut. Although the results of our work and published studies seem to argue for a MMP13-dependent contribution during the pathogenesis of chronic intestinal inflammation and intestinal fibrosis, the prognostic and/or predictive value of MMP13 levels has not been fully addressed yet. Thus, further research is needed to evaluate if MMP13 expression is suited to serve as a biomarker for the stratification of subgroups of patients with IBD, e.g. to identify individuals prone to develop fibrostenotic complications in CD.

Our work also included experiments addressing the cellular source of MMP13. In fact, we identified Pdpn+ and αSMA+ fibroblasts as origin of MMP13 in stenotic CD patients, which was in accordance with previous reports on stromal cells expressing MMP13 in IBD patients (27, 46, 47). Additional gut resident cells could also supply MMP13 during intestinal fibrogenesis, e.g. it was reported that MMP13 can be produced by macrophages and other immune cells, endothelial cells and epithelial cells in the gut including the context of IBD (27, 45, 47). Taken together, the pool of MMP13 molecules during fibrotic remodeling of the gut and intestinal inflammation is probably fed by activated intestinal fibroblasts and possibly other cell types such as macrophages.

Similar to the gene expression data in human samples, we observed that MMP13 was increased on RNA and protein level in a well-established mouse model of chronic colitis and intestinal fibrosis. Our findings add to a previous study that found elevated MMP13 expression in the gut in the context of epithelial barrier dysfunction in mouse models of sepsis and acute colitis (17), suggesting that upregulation of MMP13 in mice can occur in several disease models mimicking different intestinal pathologies *in vivo*.

Although upregulation of MMP13 seems tightly linked to inflammatory stimuli in the gut (27, 45–47), molecules that modify MMP13 expression have been incompletely characterized. By using both a gain-of-function and a loss-of-function approach, we demonstrated that MMP13 is regulated by IL36R signaling *in vivo*. In fact, we noticed a decrease of MMP13 transcripts in chronic DSS colitis upon blockade of IL36R signaling, whereas injection of IL36R ligands into otherwise unchallenged wildtype mice resulted in elevated MMP13 levels in colon tissue. To our knowledge, this is the first study reporting a direct connection between IL36R signaling and MMP13 expression *in vivo*. Our work also demonstrated a strong upregulation of MMP13 in primary colon fibroblasts upon IL36R stimulation which was consistent with the concept of fibroblasts as an important source of MMP13 in the gut. Those observations - indicating MMP13 as a target gene of IL36R signaling - are in line with previous *in vitro* studies that reported an induction of MMP13 upon IL36R activation in chondrocytes (48, 49). However, it seems possible that MMP13 expression in the gut could be further modulated by additional molecules capable of activating intestinal fibroblasts and other gut resident cells. In line with that, it was shown that cytokines such as IL1β and TNFα, basic fibroblast growth factor and a bacteria derived agonist of toll-like receptor 5 were able to stimulate MMP13 expression in mesenchymal or epithelial cells derived from extraintestinal tissues such as from the skin and the joints (50–52).

Our *in vivo* studies with gene-modified mice indicated that loss of MMP13 enzyme activity resulted in diminished fibrotic disease pathology in a well-established mouse model of intestinal fibrosis, as evidenced by reduced accumulation of ECM in the mucosa, submucosa and muscularis propria, and lower numbers of αSMA+ cells in *Mmp13-/-* mice. Whereas our data demonstrating a functional contribution of MMP13 during the pathogenesis of intestinal fibrosis *in vivo* is novel, MMP13 was connected with the modulation of fibrosis in other organs before (12). Interestingly, divergent functions were reported for MMP13 in experimental models of liver fibrosis. On the one hand, a protective role was shown demonstrating decreased accumulation of ECM and a lower number of αSMA+ cells upon overexpression of MMP13 in the chronic CCL4-induced model of liver fibrosis (53, 54). On the other hand, a pathogenic role of MMP13 was indicated showing reduced activation of hepatic stellate cells and diminished accumulation of ECM using *Mmp13*-/- mice in the bile duct ligation model (23). In a model of radiation-induced pulmonary fibrosis, pro-fibrotic qualities of MMP13 were reported, too (25). Taken together, the role of MMP13 in fibrotic diseases seems to be dependent on the particular molecular context related to the type of experimental model and organ site specifics. That is in line with a concept in which the fibrogenic role of (active) MMP-13 very much depends on its temporospatial expression, as well as the model used. Thus, most remodeling MMPs likely prepare the ground for fibrosis at acute and earlier stages, while they can promote collagen removal and fibrosis regression in non- or low inflammatory chronic scars (12). Accordingly, it has to be noted that the use of a constitutive *Mmp13-/-* mouse as employed in our study could be very different from the application of an inducible knockout system or a later stage pharmacological intervention.

Whereas MMP13 is a zinc-dependent endopeptidase with preferential activity towards type II collagen and aggrecan (13, 14), the mechanisms through which MMP13 activity influences the immune cell compartment and fibrosis are incompletely understood. In accordance with a concept viewing MMP13 as a proteolytic activator of proinflammatory non-matrix substrates such as CCL2, CCL7, TNFα and pro-MMP9 (15–17, 26), *Mmp13*-/- mice in our study showed endoscopic signs of reduced chronic colitis which was confirmed by histopathological analysis demonstrating lower numbers of CD45+ immune cells. In terms of organ fibrosis, MMP13 could influence mechanisms that exceed the regulation of inflammation, e.g. it was reported that MMP13 can influence the proliferation and activation of hepatic stellate cells and further mediate the degradation of ECM *via* activation of MMP2 and MMP9 in an experimental model mimicking fibrotic disease of the liver (24, 53). Whereas similarities to the pathogenesis in other organs seem likely for fibrotic remodeling of the gut, further studies are required to address such issues and to gain a detailed understanding of the mechanisms mediated by MMP13 activity during intestinal fibrogenesis in experimental models and human diseases.

Interestingly, findings of this study and previous work (7) demonstrated that both the inhibition of IL36R signaling and the inactivation of MMP13 were able to reduce intestinal fibrosis in the same experimental model. Mechanistic experiments of the present study further indicated an axis involving IL36R signaling and MMP13 in the intestine, suggesting that MMP13 could act as a key effector molecule downstream of IL36R activation during the development of intestinal fibrosis. However, MMP13-mediated promotion of fibrosis could also include mechanisms that occur independent from IL36R activation, which is supported by studies that connected the regulation of MMP13 expression with signaling of other pro-inflammatory cytokines frequently present at sites of intestinal inflammation such as IL1β or TNFα (50). Moreover, in view of the pleiotropic functions of IL36R signaling, IL36R-associated intestinal fibrogenesis likely includes mechanisms that happen independent from MMP13 (6, 7). Thus, future studies need to dissect the quantitative contributions of the IL36R-MMP13 axis during intestinal fibrogenesis in experimental models and their transferability to fibrostenotic CD and other diseases in humans.

Nevertheless, both IL36R and MMP13 may represent promising target structures to interfere with the development of intestinal fibrosis. Our data support efforts to inhibit IL36R signaling and/or MMP13 activity therapeutically. Of note, efficient blockade of IL36R is already available with the monoclonal antibody spesolimab, as evidenced by the therapeutic success as approved therapy in patients with generalized pustular psoriasis (GPP). Moreover, antibody-mediated inhibition of the IL-36R pathway is also studied as therapeutic concept in recent clinical trials with subgroups of patients suffering from UC (NCT03100864, NCT03123120, NCT03482635, NCT03648541) or CD (NCT03752970, NCT04362254, NCT05013385), respectively. By contrast, MMP13 inhibition to our knowledge has not found its way into clinical applications or clinical trials yet. However, multiple substances targeting MMP13 have entered the preclinical stage with different molecular strategies, e.g. zinc-binding inhibitors were developed as MMP13 activity depends on the availability of zinc ions within its catalytic site (55, 56). Small molecule inhibitors, the so-called non-zinc-binding inhibitors, target a molecular pocket of MMP13 within its catalytic site (57, 58) However, selective targeting of MMP13 seems challenging, which could be possibly related to a highly conserved 3D structure of the catalytic site among various MMPs (59). By contrast, a recent study pursued a highly specific approach and reported promising results using a strategy with nanoparticle-based Mmp13 gene silencing by siRNA (60).

Although the inhibition of MMP13 in intestinal inflammation and fibrosis appears promising from the current perspective, the optimism for approaches targeting MMPs in IBD was challenged by the results of previous clinical trials, in which a MMP9-neutralizing antibody failed to block intestinal inflammation in patients with moderately to severely active CD or UC, respectively (61, 62). Hence, additional studies are required to further evaluate the therapeutic potential of targeting IL36R-inducible MMP13 in intestinal inflammation and fibrosis.

## Data availability statement

The datasets presented in this study can be found in online repositories. The names of the repository/repositories and accession number(s) can be found below: https://www.ebi.ac.uk/biostudies/arrayexpress/: E-MTAB-6476, E-MTAB-12788.

## Ethics statement

The studies involving human participants were reviewed and approved by ethical review committee of Friedrich-Alexander University Erlangen-Nürnberg. The patients/participants provided their written informed consent to participate in this study. The animal study was reviewed and approved by Regierung von Unterfranken. Written informed consent was obtained from the owners for the participation of their animals in this study.

## Author contributions

KK, KE, MH, LR, MV, GS, ZT, AK, RA, ML, PB, JR, AM, MN, CN provided protocols or samples or designed the experiments or analysed sequencing data; KK, KE, MH, LR performed the experiments; KK, MN, CN analyzed, discussed and interpreted data; KK and CN wrote the manuscript. All authors contributed to the article and approved the submitted version.

## Supplementary material

Supplementary Figure 1 The expression levels of the 20 highest expressed DEGs were plotted against the active inflammation during endoscopy. The endoscopic disease severity (none=0, low=1, medium=2, high=3) of all 16 samples from the stenosis cohort was plotted against the log2CPM (counts per million) of the 20 highest differentially expressed genes with decreasing order by their log2 fold change (referring to **Figure 1E** and **Supplementary Table 1**).

Supplementary Figure 2 The gene expression levels of the 20 highest expressed DEGs are depicted in relation to the disease duration. The disease duration (in years) of the 8 patients from the stenosis cohort was plotted against the gene expression in the stenosis samples. The gene expression of the 20 highest differentially expressed genes in log2CPM (counts per million) with decreasing order by their log2 fold change (referring to **Figure 1E** and **Supplementary Table 1**) is depicted.

Supplementary Figure 3 The induction of Mmp13 expression in WT mice upon IL36R ligand injection is dose dependent. Wildtype mice were injected i.p. with 0.1 µg, 0.5µg and 2.5µg or PBS as control (n=5 in each group). At the next day, colon tissue was harvested and used for RNA isolation. The MMP13 expression was measured by qPCR. Quantitative data were analyzed by one-way ANOVA followed by Dunnett’s multiple comparisons test (***p>0.001) and mean values are shown with standard deviation.
