# Supplementary material for: Interleukin 36 receptor-inducible matrix metalloproteinase 13 mediates intestinal fibrosis
Source: Front Immunol. 2023 May 3;14:1163198. doi: 10.3389/fimmu.2023.1163198 (PMC10189878; doi:10.3389/fimmu.2023.1163198)
Supplement: Supplementary file 1 [file Table_1.pdf]

n	8	
Mean age, y (range)	53 (36-78)	
Females	5	
Males	3	
Mean disease duration, y	18 (6-36)	
Localization		
Caecum	1	
Colon ascendens	2	
Colon transversum	2	
Sigma	3	
Rectum	0	
Inflammatory activity during endoscopy	Healthy area	Stenotic area
no	8 (100%)	0
low	0	4 (50%)
medium	0	1 (12.5%)
high	0	2 (25%)
N.A.	0	1 (12.5%)

Supplemental Table 1. Characteristics of CD patient samples of the stenosis cohort
