# Supplementary material for: Interleukin 36 receptor-inducible matrix metalloproteinase 13 mediates intestinal fibrosis
Source: Front Immunol. 2023 May 3;14:1163198. doi: 10.3389/fimmu.2023.1163198 (PMC10189878; doi:10.3389/fimmu.2023.1163198)
Supplement: Supplementary file 2 [file Table_2.pdf]

	non-IBD	CD: B1 (non-stricturing, non-penetrating)	CD: B2 (stricturing)
n	51	45	22
Mean age, y (range)	59.3 (22-84)	39.9 (21-70)	37.8 (22-74)
Mean disease duration, y (range)	/	2.3 (1-4)	14.1 (10-27)
Females	24	29	15
Males	27	15	7
N.A.	0	1	0
Origin of biopsy			
Ileum	29	34	16
Colon	2	11	6

Supplemental Table 2. Characteristics of samples from the IBDome cohort
