# Supplementary material for: Interleukin 36 receptor-inducible matrix metalloproteinase 13 mediates intestinal fibrosis
Source: Front Immunol. 2023 May 3;14:1163198. doi: 10.3389/fimmu.2023.1163198 (PMC10189878; doi:10.3389/fimmu.2023.1163198)
Supplement: Supplementary file 3 [file Table_3.pdf]

	Uninflamed control	CD patient with stenosis
n	11	8
Mean age, y (range)	32.2 (18-50)	48.9 (29-68)
Mean disease duration, y	5.9	6.3
Females	5	3
Males	6	5
Localization		
Caecum	1	1
Colon ascendens	3	0
Colon transversum	4	1
Sigma	1	5
Rectum	2	1
Active inflammation (tissue section)	0	8

Supplemental Table 3. Characteristics of samples of the IF cohort
