# Supplementary figures and images for: Interleukin 36 receptor-inducible matrix metalloproteinase 13 mediates intestinal fibrosis
Source: Front Immunol. 2023 May 3;14:1163198. doi: 10.3389/fimmu.2023.1163198 (PMC10189878; doi:10.3389/fimmu.2023.1163198)

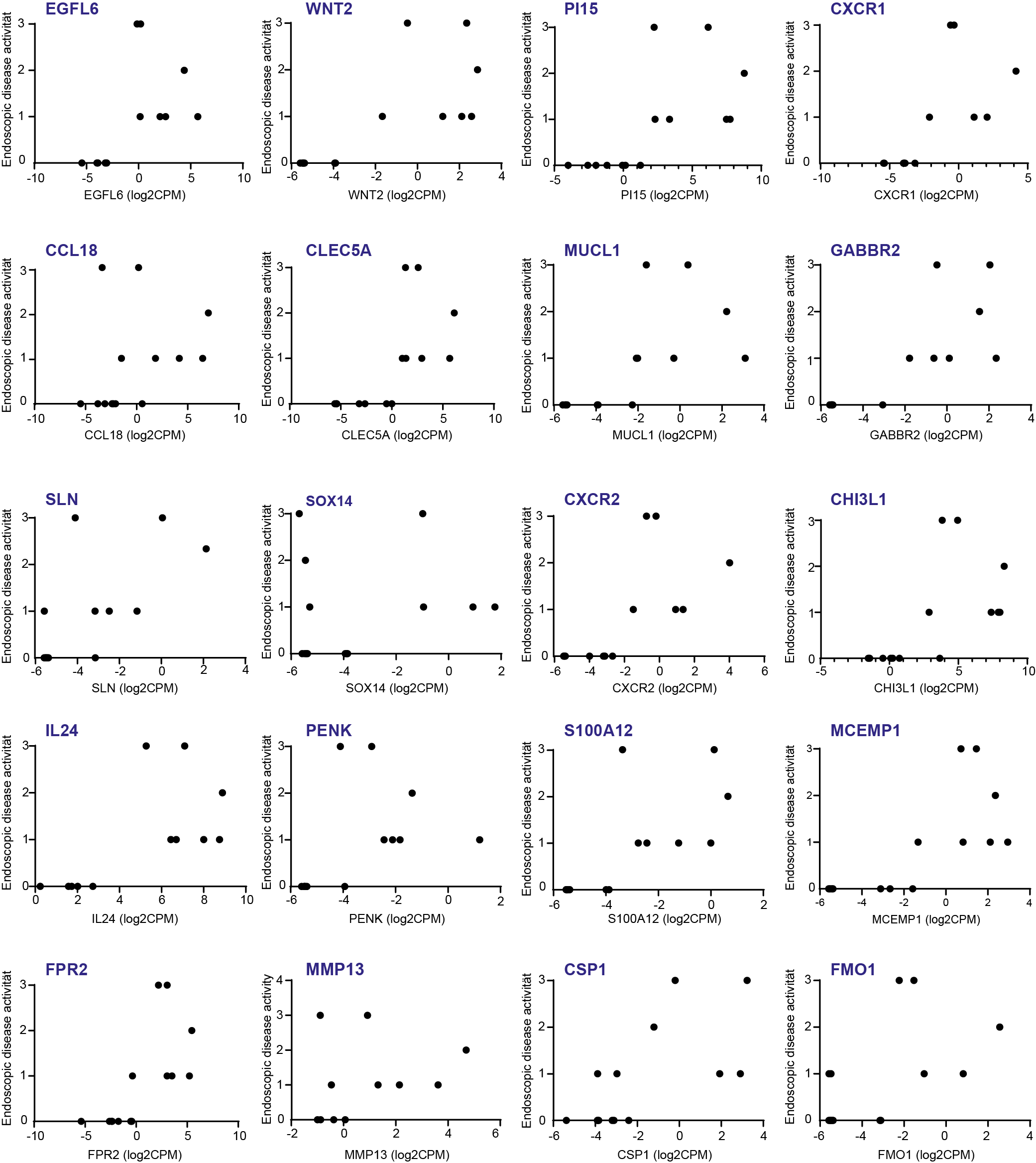

Supplement: Supplementary Figure 1 — The expression levels of the 20 highest expressed DEGs were plotted against the active inflammation during endoscopy. The endoscopic disease severity (none=0, low=1, medium=2, high=3) of all 16 samples from the stenosis cohort was plotted against the log2CPM (counts per million) of the 20 highest differentially expressed genes with decreasing order by their log2 fold change (referring to Figure 1E and Supplementary Table 1 ). [file Image_1.tif]

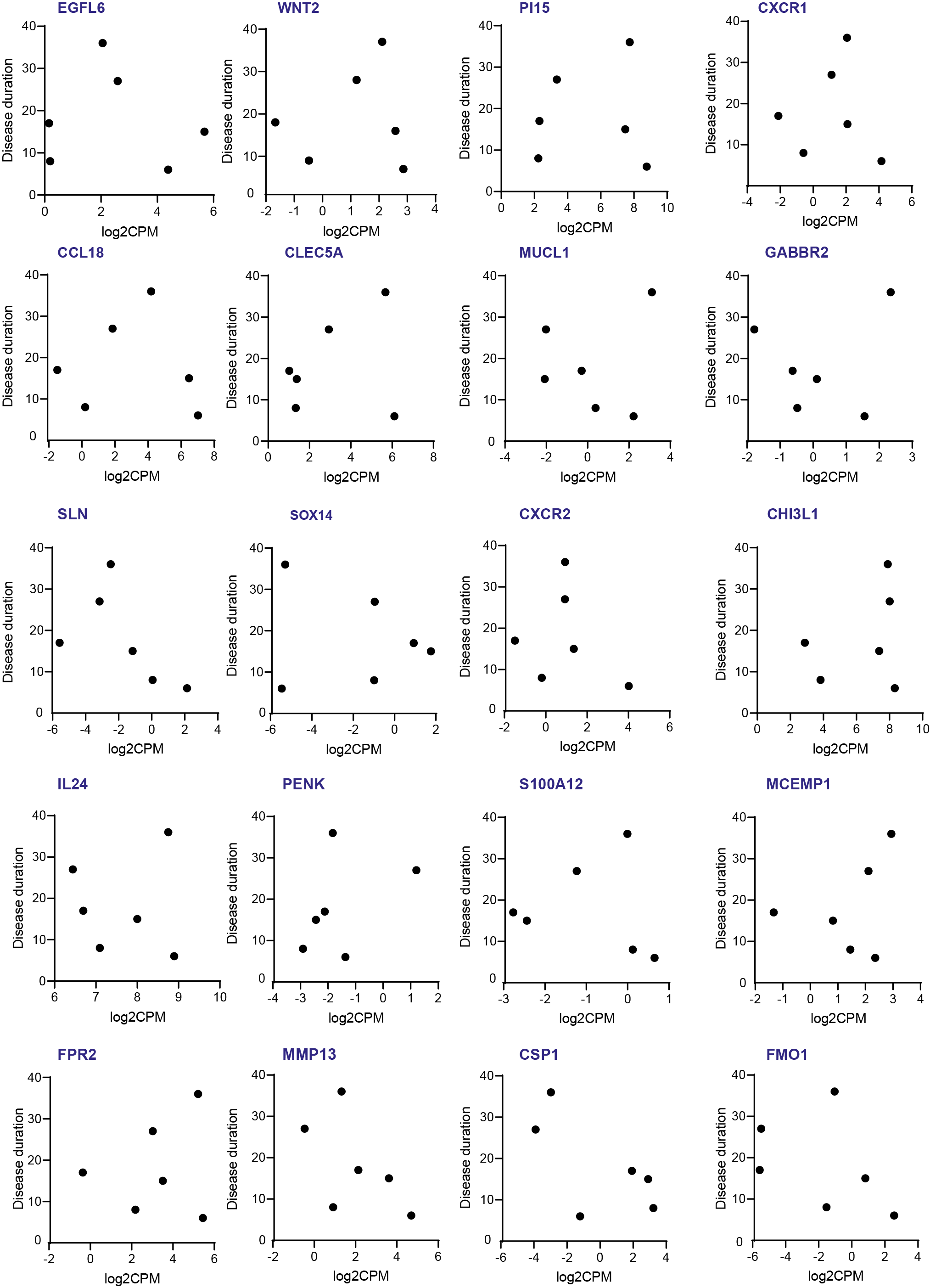

Supplement: Supplementary Figure 2 — The gene expression levels of the 20 highest expressed DEGs are depicted in relation to the disease duration. The disease duration (in years) of the 8 patients from the stenosis cohort was plotted against the gene expression in the stenosis samples. The gene expression of the 20 highest differentially expressed genes in log2CPM (counts per million) with decreasing order by their log2 fold change (referring to Figure 1E and Supplementary Table 1 ) is depicted. [file Image_2.tif]

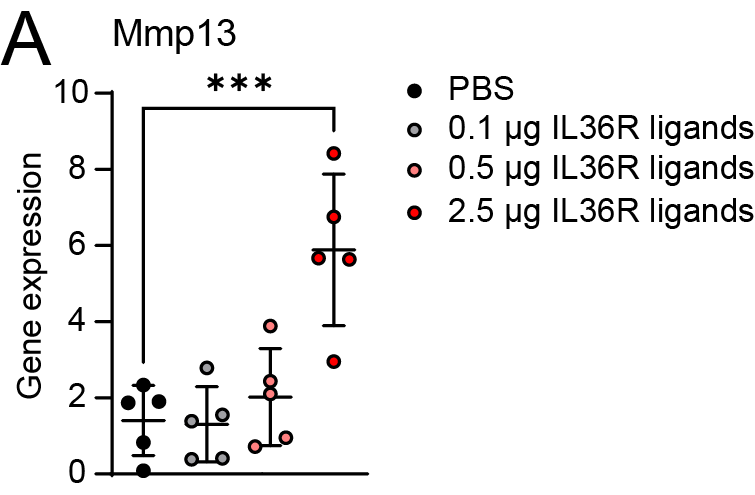

Supplement: Supplementary Figure 3 — The induction of Mmp13 expression in WT mice upon IL36R ligand injection is dose dependent. Wildtype mice were injected i.p. with 0.1 µg, 0.5µg and 2.5µg or PBS as control (n=5 in each group). At the next day, colon tissue was harvested and used for RNA isolation. The MMP13 expression was measured by qPCR. Quantitative data were analyzed by one-way ANOVA followed by Dunnett’s multiple comparisons test (***p>0.001) and mean values are shown with standard deviation. [file Image_3.tif]
